# Computational phenotyping of effort-based decision-making in type-2 diabetes on and off semaglutide

**DOI:** 10.1038/s41386-026-02433-y

**Published:** 2026-05-06

**Authors:** Sara Z. Mehrhof, Hugo Fleming, Camilla L. Nord

**Affiliations:** 1 MRC Cognition and Brain Sciences Unit, University of Cambridge, Cambridge, UK; 2 Department of Psychiatry, University of Cambridge, Cambridge, UK

**Keywords:** Motivation, Human behaviour, Decision, Depression

## Abstract

Motivation plays a fundamental role in human behaviour. Dopaminergic pathways have long been implicated in individual differences in motivation. Emerging evidence suggests such neural mechanisms interact with metabolic processes to coordinate energy expenditure with energy resources, thereby linking motivation with metabolic health. We ask whether a cognitive-computational index of motivation—reliably linked to neuropsychiatric symptoms—is altered in the context of type-2 diabetes and treatment with a GLP-1 agonist (semaglutide). In a pre-registered experiment, we quantified computational effort-based decision-making parameters in participants with diabetes on (*N* = 58) or off (*N *= 54) semaglutide treatment, compared to two groups of matched controls without diabetes (*N* = 58 each). Subjects with type-2 diabetes showed a blunted *acceptance bias*, a computational parameter describing the bias to accept effort for reward. This effect was not driven by neuropsychiatric comorbidity or antidepressant use. Across all participants, we found that increasing diabetes risk linearly predicted reduced acceptance bias. Participants with diabetes treated with semaglutide did not show restored motivation. Metabolic ill-health is associated with reduced acceptance bias during motivational decision-making. This blunting mirrors—but is largely independent of—neuropsychiatric motivational deficits. This suggests metabolic ill-health is accompanied by a cognitive shift towards energy conservation, potentially contributing to comorbidity between metabolic ill-health and mental illness.

## Introduction

Motivation underpins every facet of human behaviour. Higher motivation is associated with better outcomes across numerous domains, including life satisfaction [1], while reduced motivation (variously termed anhedonia, apathy or avolition) is a key transdiagnostic symptom of neurological and psychiatric conditions [2].

Empirical work suggests a neurocognitive mechanism underpins motivational differences: the integration of costs and benefits during decision-making (effort-based decision-making) [3, 4]. For such decision-making to be adaptive, effort costs and benefits associated with an action need to be coordinated with internal energy resources. Allostatic theories propose that the brain dynamically regulates energy allocation based on physiological needs and demands [5–7]; cognitive processes underlying motivation could represent a regulatory mechanism aligning behaviour with metabolic needs [8]. Hence, the delicate balance between expending effort to obtain resources and energy conservation closely ties motivational decision-making to our energy metabolism, and with that, our metabolic health.

Metabolic disorders like type-2 diabetes are highly comorbid with neuropsychiatric disorders, and motivational symptoms are postulated to play a mediating role in the relationship [9]. An overall shift towards greater energy conservation, manifesting partly as reduced effort expenditure, could constitute a common mechanism linking mental and metabolic health. This points to the existence of an overarching energy regulation phenotype spanning cognitive and physical health disorders [10, 11]. However, individual differences in metabolic health have seldom been considered when investigating the neurocognitive mechanism of motivation.

Convergent evidence linking motivation with metabolic processes stems from the neurobiology of motivation. Motivation depends primarily on dopaminergic signalling in the brain, particularly involving projections from the ventral tegmental area (VTA) to the ventral striatum [2]. Neurons along this pathway also express receptors for insulin, which typically increases dopamine release [12]. In turn, as well as enhancing motivation, striatal dopamine has a number of effects relevant to energy homeostasis, including inhibiting appetite and suppressing endogenous glucose production [13]. Conversely, insulin insensitivity may disrupt dopaminergic signalling and motivation. Both animal and human studies have shown insulin resistance alters dopaminergic signalling in the nucleus accumbens [14, 15] and comorbid metabolic factors are shown to exacerbate motivational deficits in major depressive disorder [16].

However, it remains unclear whether poor metabolic health alone is associated with blunted motivational decision-making and whether the effect of metabolic health on motivational decision-making is parallel, distinct, or overlapping with effects of neuropsychiatric symptoms. Moreover, it remains to be investigated how effective metabolic treatments affect motivational decision-making. In this regard, GLP-1 receptor agonists like semaglutide may be of particular interest, due to their putative direct effects on cognition [17], as well as its rapid growth in clinical use and prescription. To test this, we investigated whether a cognitive-computational signature of motivation—reliably linked to neuropsychiatric symptoms, including anhedonia and apathy—was affected by metabolic ill-health and treatment. Moreover, we assessed effects of semaglutide treatment on psychiatric and behavioural questionnaire-based measures. We hypothesised that people with type-2 diabetes would exhibit blunted motivation to exert effort for reward, and that this would be restored in patients undergoing semaglutide treatment.

## Methods

The current study was approved by the University of Cambridge Psychology Research Ethics Committee (PRE.2022.078). All participants provided informed consent through an online form, complying with procedures for online studies. Our pre-registration can be found at osf.io/7kmf5. Analysis code and data are available at github.com/smehrhof/semaglutide-study.

### Participants

**Fig. 1:**
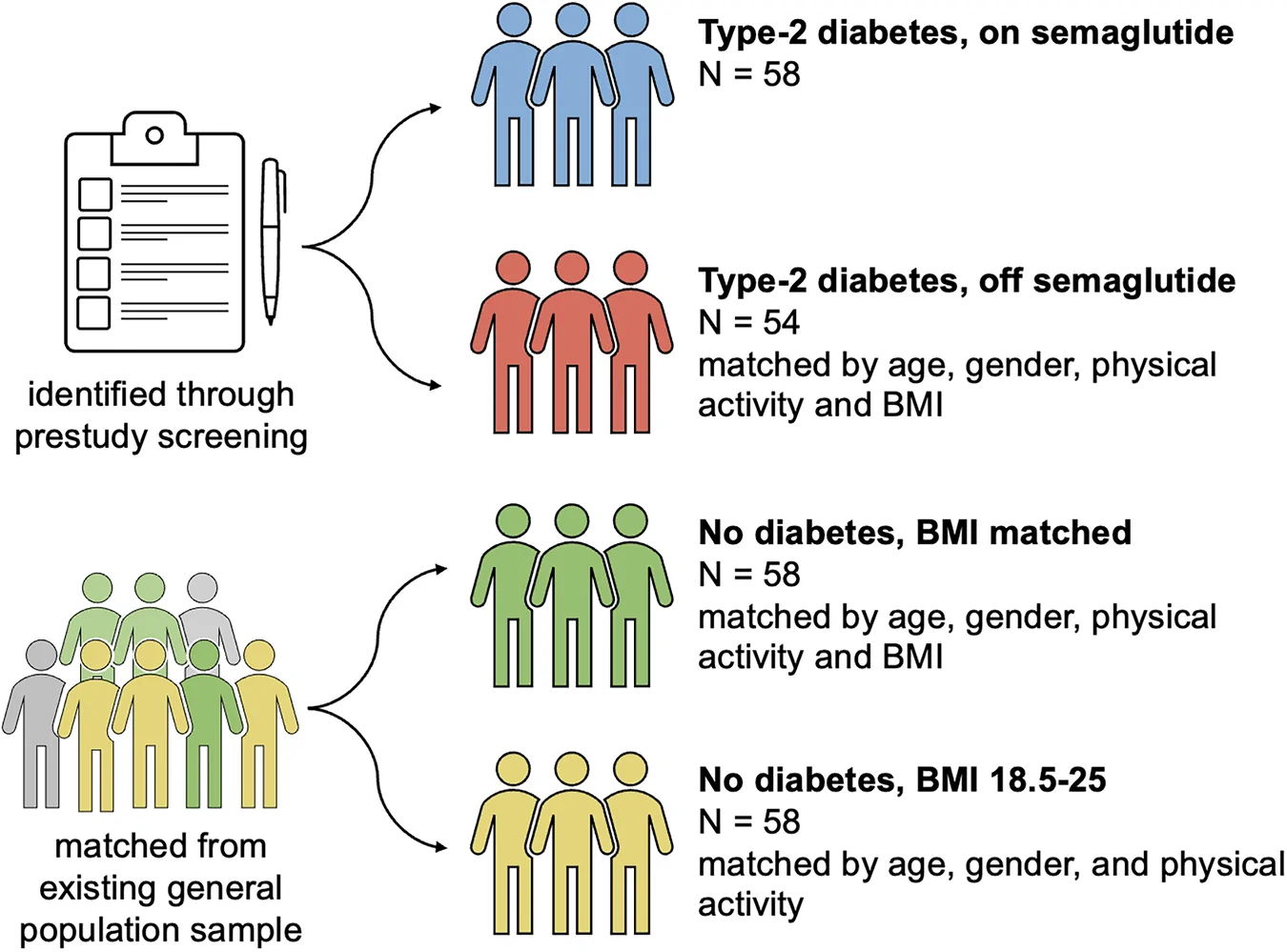
Study groups. Our sample consisted of four study groups. Two groups of participants with type-2 diabetes (differing in their current semaglutide treatment status) were identified through a pre-study screening. Two groups of control participants with no diabetes (differing in their body mass index [BMI]) were matched to the type-2 diabetes groups from a preexisting general population sample.

Our study includes four study groups, differing in metabolic health and medication status (Fig. 1):

1. ***Type-2 diabetes, on semaglutide:*** Participants with type-2 diabetes, treated with semaglutide currently (and for at least the past 4 weeks).
2. ***Type-2 diabetes, off semaglutide:*** Participants with type-2 diabetes and no current or past GLP-1 agonist treatment; matched to type-2 diabetes*, on semaglutide* by age, gender, physical activity, and body mass index (BMI)

1. ***No diabetes, BMI matched:*** Participants without diabetes and no current or past GLP-1 agonist treatment; matched to *type-2 diabetes, on semaglutide* by age, gender, physical activity, and BMI; Finnish Type-2 Diabetes Risk Score (FINDRISC) [18] < 12 (slight metabolic risk)
2. ***No diabetes, BMI 18.5–25:*** Participants without diabetes and no current or past GLP-1 agonist treatment; matched to *type-2 diabetes, on semaglutide* by age, gender, and physical activity; BMI between 18.5 and 25; FINDRISC < 7 (low metabolic risk)

### Recruitment and data acquisition

All data was collected on Prolific [19] in the United States and the United Kingdom.

To recruit participants with type-2 diabetes, we implemented a screening procedure including demographics (age, gender, ethnicity, socio-economic status, income, and English proficiency), general health (neurological conditions, mental health/neurodevelopmental conditions, chronic disease, and daily medication), current GLP-1 receptor agonist treatment status, metabolic health (BMI, other current treatments for diabetes), and physical activity (International Physical Activity Questionnaire—short form (IPAQ) [20]). Participants indicating current GLP-1 agonist treatment were asked for treatment details (type of GLP-1 agonist, mode and schedule of administration, dosage).

Participants fulfilling criteria to be included in the *type-2 diabetes, on semaglutide* group were invited to the main testing. Subjects indicating, they are not currently (nor have ever been) on any form of GLP-1 agonist treatment were matched to the *type-2 diabetes, on semaglutide* group by age, gender, BMI, and IPAQ and invited to the main testing.

To obtain control groups of participants without diabetes, we used a pre-existing general population dataset from a previous study, implementing a largely overlapping procedure [21]. Participants without diabetes not treated with any GLP-1 agonist were identified. *No diabetes, BMI matched* subjects were matched to the *type-2 diabetes, on semaglutide* group by age, gender, IPAQ, and BMI. *No diabetes, BMI 18.5–25* subjects were matched to the *type-2 diabetes, on semaglutide* group by age, gender, and IPAQ.

### Procedure

The main testing session consisted of three parts: assessment of medication changes since the screening, an effort-based decision-making task, and a battery of self-report questionnaires.

#### Effort-based decision-making task

**Fig. 2:**
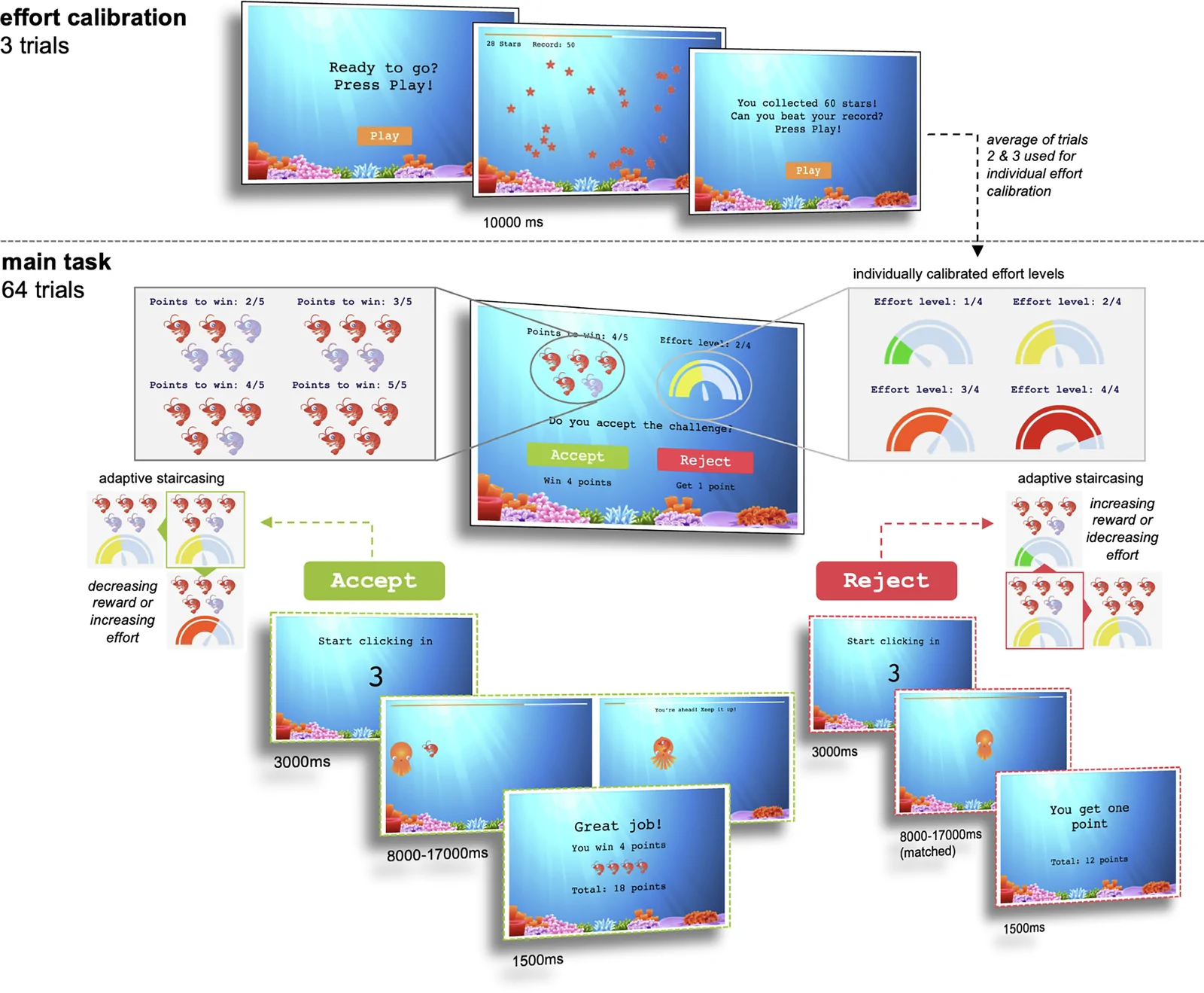
Effort-based decision-making task. The task comprised an effort calibration phase followed by the main task. During calibration, participants completed three trials in which they clicked as fast as possible for 10 s; the mean of trials 2 and 3 was used to set each participant’s individual effort threshold. The main task consisted of 64 trials. On each trial, participants were presented with an offer specifying a reward (2, 3, 4, or 5 points) and an effort level (1–4) and chose to accept or reject it. Accepted offers required participants to complete a physical challenge at the specified effort level to earn the offered points; rejected offers resulted in a fixed waiting period of matched duration and a reward of 1 point.

We used a previously described effort-expenditure task [21] to assess effort-based decision-making (Fig. 2). The task starts with a calibration phase in which subjects are prompted to click as fast as they can for ten seconds, repeated across three trials. The average of trials two and three are used as a reference for effort calibration. Specifically, effort levels are scaled to a participant’s clicking capacity reference and the time clicking must be sustained. We implement four effort levels, corresponding to a clicking speed at 30% of a participant’s maximal capacity for 8 s (level 1), 50% for 11 s (level 2), 70% for 14 s (level 3), and 90% for 17 s (level 4). To familiarize subjects with their individual effort levels, a practice trial of each effort level must be completed. If an effort level is failed during the practice round, it is repeated. If a subject fails a level twice, the clicking capacity reference is adjusted to the speed reached in the practice trial. This ensures that all participants experienced success at each effort level and hence knew they were able to complete each effort level, therefore avoiding any confounding of effort discounting effects by risk discounting.

The main task consists of 64 trials, split into four blocks. In each trial, participants decide whether to accept or reject a challenge associated with effort and reward. Reward is conceptualized as points, varying between four levels (2, 3, 4, or 5 points). When accepting a challenge, the associated effort level must be completed to obtain the reward. Reward receipt is solely based on successful completion of the required effort, with no associated probabilistic uncertainty. If a challenge is rejected, subjects wait and receive one point. Importantly, waiting times are matched to the respective effort level to prevent any confounding with delay discounting.

The presentation of effort-reward combinations is made semi-adaptively by randomly interleaved staircases. Each of the 16 possible offers (i.e., 4 effort levels x 4 reward levels) serves as the starting point of one staircase. Within each staircase, after a subject accepts a challenge, the next trial’s offer on that staircase is adjusted by increasing effort or decreasing reward. After a subject rejects a challenge, the next offer on that staircase is adjusted by decreasing effort or increasing reward. This ensures participants receive each effort-reward combination at least once while individualizing trial presentation to maximize each trial’s informative value.

#### Self-report questionnaires

Self-report questionnaires were presented in randomized order and, in all study groups, included the Snaith-Hamilton Anhedonia Rating Scale (SHAPS) [22], the Apathy Evaluation Scale (AES) [23], the FINDRISC [18]. In the two *type-2 diabetes* groups, but not the *no diabetes*, control groups, questionnaire-based measures further included the Beck Depression Inventory (BDI) [24], the Obsessive-Compulsive Inventory – Revised (OCI-R) [25], the Monetary Choice Questionnaire (MCQ) [26], Desire for Alcohol Questionnaire (DAQ) [27], the Munich Chronotype Questionnaire (MCTQ) [28], the Morningness-Eveningness Questionnaire (MEQ) [29], and the Three-factor Eating Questionnaire (TFEQ) [30].

#### Compliance checks and exclusion criteria

As per pre-registration, participants were excluded (at screening) when reporting a severe neurological disorder or English proficiency below B2 (i.e., good command/working knowledge). Four attention check questions were included in the questionnaires, including two easy and two harder questions. Participants were excluded when failing at least one easy, or both harder questions (*n* = 3).

Additionally, we excluded participants reporting conflicting data between the screening and main testing (*n* = 19), and those reporting a BMI above two standard deviations of the median (*n* = 5). The decision to apply these non-pre-registered exclusions was made prior to other analyses. Overall, *n* = 13 participants on semaglutide and *n* = 12 participants off semaglutide were excluded.

#### Within-subject study

A subset of participants in the *type-2 diabetes, on semaglutide* group were randomized to be tested on different days: one day after their weekly semaglutide injection (when treatment concentration is highest) or one day before (i.e., six days after their injection, when treatment concentration is reduced by half [31]). Subjects were then invited to a second study session, at the respective other day in their treatment, including the effort-based decision-making task, as well as the SHAPS, DAQ, and TFEQ. *N* = 25 participants completed two study sessions.

### Analyses

Prior to computational modelling, we used model-agnostic analyses to ensure our task elicited effects of effort discounting across groups. We investigated main effects of effort- and reward-levels and their interaction, using a mixed-effects analysis of variances (ANOVA) of repeated measures.

For model-based analyses, we considered a model space of six models, all of which are variations on a standard decision-theory model. In brief, rewards (R) are discounted by effort (E), weighted by sensitivity parameters (${\beta }_{R}$ and ${\beta }_{E}$ for reward and effort sensitivity), forming the subjective value (SV) of an action:

1 $${SV}=\left({\beta }_{R}\cdot R\right)-({\beta }_{E}\cdot E)$$

Higher effort and reward sensitivity mean the SV is more strongly influenced by changes in effort and reward, respectively. The SV is transformed to an acceptance probability by a softmax function:

2 $$p\left({accept}\right)=\frac{1}{1+\,{e}^{-(\alpha \,+\,{SV})}}$$

with $p({accept})$ for the predicted acceptance probability and α for the intercept representing acceptance bias. A higher acceptance bias means one has an overall increased tendency, or bias, to accept rather than reject offers.

Models differed from each other in their implemented discounting function (parabolic or linear) and the in- or exclusion of free parameters reward sensitivity (weighting of the reward magnitude during decision making) and acceptance bias (decision bias towards exerting effort), resulting in a model space of six models (parabolic/linear x with/without reward sensitivity x with/without acceptance bias).

We took a hierarchical Bayesian approach to model fitting, using weakly informative Gaussian priors for all free parameters, at both the group- and individual-level. Models were fitted in the R Stan interface cmdstanr, using Markov-Chain Monte Carlo (MCMC) sampling (6000 iterations by four chains, 2000 warm-up samples) [32]. Model performance was compared by out-of-sample predictive accuracy using the leave-one-out cross-validation information criterion (LOOIC; lower values are better).

A previous validation study of the task and modelling approach demonstrated good parameter recovery (Pearson’s correlations between underlying and recovered parameters >0.8 for all models and parameters), and no spurious correlations between parameters [21].

Group comparisons were conducted using one-way ANOVAs, with group status predicting computational parameter estimates and questionnaire scores. To follow up on significant effects, multiple comparisons were conducted.

We tested the effect of time since the last semaglutide injection on computational parameters using paired sample t-tests, comparing testing sessions one and six days after the last semaglutide injection.

## Results

The four study groups did not differ in age, gender, physical activity, and, for the first three groups, BMI, validating our matching procedure (Table 1). Effort-discounting effects during the task were confirmed in all groups (main effects of effort and reward on acceptance probability *p* < 0.001). Further model agnostic analyses of task data are provided in the Supplemental Information Section 1.

**Table 1:** Demographic characteristics of the sample, by study group.

	Type-2 diabetes, on semaglutide	Type-2 diabetes, off semaglutide	No diabetes, BMI matched	No diabetes, BMI 18.5–25
Sample size, number (%)	58 (25.44)	54 (23.68)	58 (25.44)	58 (25.44)
Demographics				
Age, mean (SD, range)	44.7 (13.09; 19-74)	45.9 (11.37; 25-68)	48.3 (13.58; 21-69)	45.4 (14.33; 21-74)
Gender, number (%)				
Female	31 (53.45)	34 (62.96)	28 (48.28)	40 (68.97)
Male	26 (44.83)	20 (37.04)	30 (51.72)	17 (29.31)
Non-binary	1 (1.72)	0 (0)	0 (0)	1 (1.72)
BMI, mean (SD)	37.6 (8.53)	37.8 (10.0)	35.0 (6.60)	22.3 (1.80)
Current or past psychiatric disorder, number (%)	41 (70.70)	31 (57.41)	12 (20.69)	15 (25.86)
Physical activity				
IPAQ, mean sum score (SD)	2403 (4185)	3363 (4987)	2516 (2272)	2887 (2582)
Psychiatric questionnaires				
AES, mean sum score (SD)	54.4 (10.7)	55.1 (11.0)	56.3 (9.52)	57.3 (8.52)
SHAPS, mean sum score (SD)	10.4 (7.71)	8.83 (6.73)	9.76 (6.82)	8.31 (6.27)

*BMI* body mass index, *SD* standard deviation, *IPAQ* International Physical Activity Questionnaire (sum scores reflect total *Metabolic Equivalent of Task (MET) minutes* per week), *AES* apathy evaluation scale, *SHAPS* Snaith-Hamilton Pleasure Scale.

All models included in our model-based analysis converged, and formal model comparison indicated best fit for a parabolic discounting function and inclusion of all three free parameters, in line with previous studies. Posterior predictive checks further validated the winning model. See Supplementary Information Section 2 for full computational modelling validation results.

### Type-2 diabetes subjects show a reduced bias to accept effort for reward

**Fig. 3:**
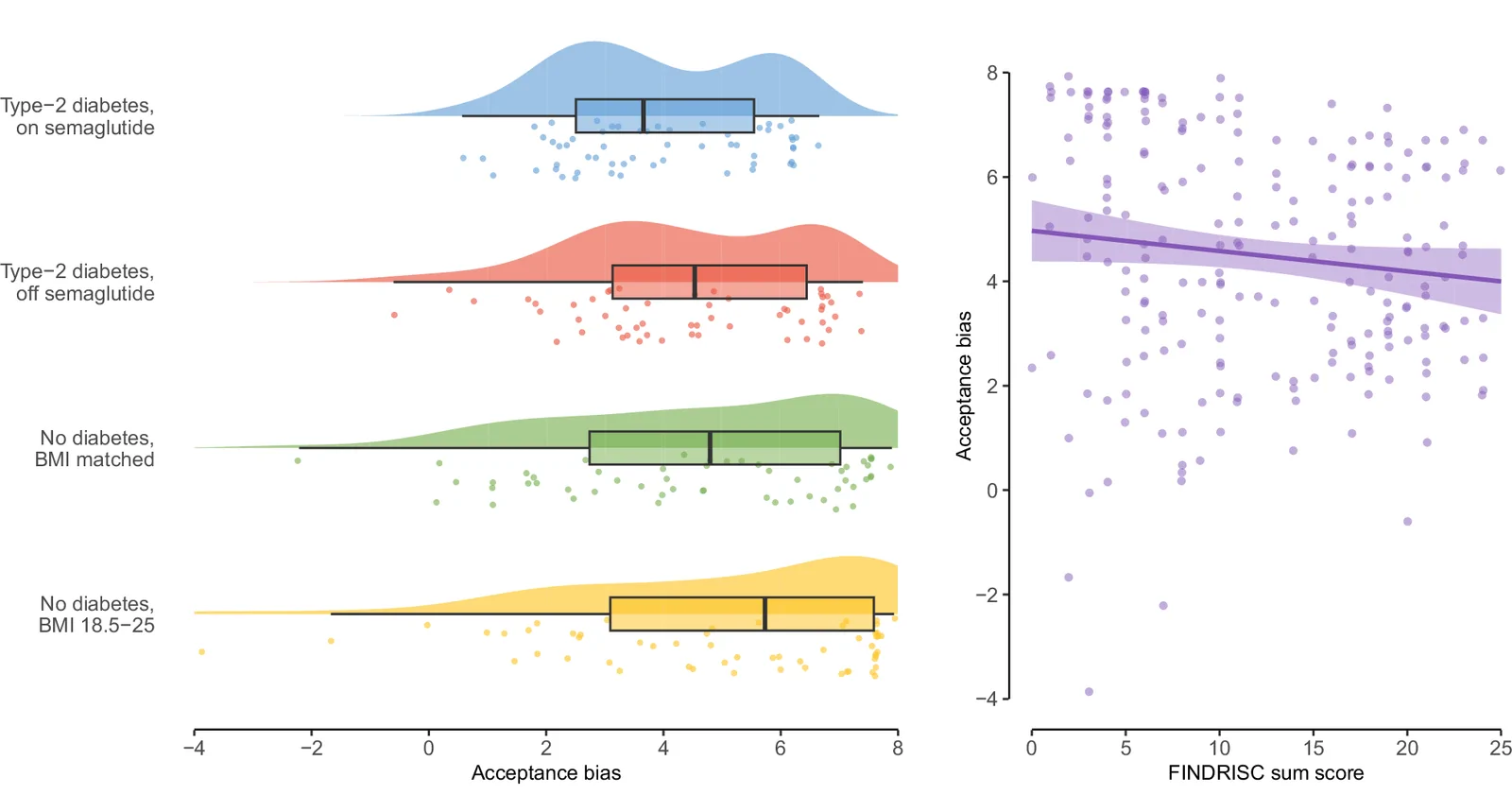
Metabolic effects on the bias to accept effort for reward. Left: Comparing individual-level parameter estimates of acceptance bias between four study groups. Right: Dimensional effect of diabetes risk, assessed with the Finnish diabetes risk score (FINDRISC) on individual-level parameter estimates of acceptance bias.

In our preregistered analyses, we found a main effect of group on acceptance bias (*F*(3, 224) = 5.025, *p* = 0.002), such that participants in the *no diabetes, BMI 18.5–25* group had the highest acceptance bias to exert effort for rewards, and participants in the *type-2 diabetes, on semaglutide* the lowest (Fig. 3, left panel). Post-hoc comparisons show the effect was driven by differences between *type-2 diabetes, on semaglutide* and both the *no diabetes, BMI matched* (*t*(90.501) = 2.451, *p* = 0.016) and *no diabetes, BMI 18.5–25* participants (*t*(87.572) = 3.803, *p* < 0.001), as well as the difference between *type-2 diabetes, on semaglutide* and *no diabetes, BMI 18.5–25* (*t*(100.08) = 2.309, *p* = 0.023). Note that only the effect between *type-2 diabetes, on semaglutide* and *no diabetes, BMI 18.5–25* remained significant after Bonferroni correction.

There was no group effect on effort (*F*(3, 224) = 0.60, *p* = 0.62) or reward sensitivity (*F*(3, 224) = 1.38, *p* = 0.25).

We also tested if our main group effect was robust to antidepressant use and current/past psychiatric disorder, in order to examine if they were driven entirely by psychiatric burden. There were between-group differences in antidepressant use (highest in *type-2 diabetes, on semaglutide*, lowest in the two *no diabetes* groups; *X*^2^(3) = 25.191, *p* < 0.001, Table 1), and current or past psychiatric disorder (highest in *type-2 diabetes, on semaglutide*, lowest in *no diabetes, BMI matched*; *X*^2^(3) = 41.33, *p* < 0.001, Table 1), but not neuropsychiatric symptom scores (AES: *F*(3, 224) = 0.555, *p* = 0.645; SHAPS: *F*(3, 224) = 1.212, *p* = 0.306, Table 1). Nevertheless, the group effect on acceptance bias was robust to controlling for antidepressant use (group effect: *F*(3, 223) = 5.017, *p* = 0.002; antidepressant effect: *F*(1, 223) = 0.632, *p* = 0.428) and current or past psychiatric disorder (group effect: *F*(3, 223) = 5.039, *p* = 0.002; current/past psychiatric disorder effect: *F*(1, 223) = 1.624, *p* = 0.204).

### An increasing risk for diabetes linearly predicts reduced acceptance bias

Due to our main effect of group, but inconsistent effects on linear contrasts, we also tested the linear relationship between acceptance bias and metabolic risk score (FINDRISC) across all groups in a generalized linear model. While the FINDRISC is primarily used as a screening tool for individuals at risk for diabetes [18], its items targeting body composition, dietary, and lifestyle habits make it an applicable tool for quantifying metabolic health across individuals with and without diabetes. Metabolic risk significantly predicted acceptance bias *b* = –0.129, *t* = -–2.489, *p* = 0.014; (Fig. 3 right panel). Again, the effect remained significant when controlling for antidepressant use (*b* = –0.11, *t* = –2.01, *p* = 0.046). However, when controlling for current or past psychiatric disorders, in this instance, the effect was only marginally significant (*b* = –0.10, *t* = –1.78, *p* = 0.08), presumably explained by the high multicollinearity (with current/past psychiatric disorders: FINDRISC *M* = 14.14, *SD* = 6.85; without current/past psychiatric disorders: FINDRISC *M* = 9.77, *SD* = 6.57; *t(206.38)* = 5.17, *p* < 0.001).

### Current semaglutide medication did not affect effort-based decision-making

Contrary to our hypotheses, we found no difference on computational parameters between the matched *on* and *off semaglutide* type-2 diabetes groups (acceptance bias: *t(104.38)* = 1.50, *p* = 0.14; effort sensitivity: *t(110)* = 0.99, *p* = 0.33; reward sensitivity: *t(97.54)* = 0.02, *p* = 0.99). Moreover, the *type-2 diabetes, on semaglutide* and *type-2 diabetes, off semaglutide* groups did not differ in questionnaire-based measures (*p* > 0.05 for all questionnaires, see Supplemental Information Section 3 for a full description of questionnaire-based measures).

To further contextualize these null findings and explore whether this may be due to sampling biases between the groups, we conducted exploratory descriptive analyses examining proxies for disease severity across the two type-2 diabetes groups. We considered the number of diabetes medication classes, number of diabetes relevant physical comorbidities, and cardiovascular medication burden. Results indicated that the *type-2 diabetes, on semaglutide* group exhibited more advanced disease progression, as reflected by a greater number of diabetes medication classes, a more comorbidities, and a higher cardiovascular medication burden (see Supplemental Information Section 4 for further information).

Given the high proportion of participants in the type-2 diabetes groups currently treated with antidepressant medication, particularly in the *on semaglutide* group (*on semaglutide*: 30 participants (51.724%) on antidepressants; *off semaglutide*: 16 participants (29.630%) on antidepressants), we next examined the association between antidepressant use and the acceptance bias across all participants with type-2 diabetes. We found no association between antidepressant use and the acceptance bias parameter (*t*(110) = –0.400, *p* = 0.690).

### Time since semaglutide injection did not affect effort-based decision-making

Within the *type-2 diabetes, on semaglutide* group, time since injection did not affect decision-making (within-subjects comparison of one to six days since injection, *n* = 25: *t*(24) = 0.237, *p* = 0.814), or questionnaire-based measures (SHAPS: *t*(24) = –0.03, *p* = 0.98; DAQ: *t*(24) = 0.21, *p* = 0.83; TFEQ Cognitive Restrained: *t*(24)=-0.47, *p* = 0.64; TFEQ Uncontrolled Eating: *t*(24) = 0.00, *p* = 1.00; TFEQ Emotional Eating: *t*(24) = –0.12, *p* = 0.90).

## Discussion

By applying approaches from computational neuropsychiatry to the metabolic domain, we found that poor metabolic health may be associated with altered effort-based decision-making. This was the case in our pre-registered primary analysis (where we report a significant group effect across groups of participants with type-2 diabetes, on and off semaglutide, and BMI-matched and -unmatched healthy controls, which numerically show decreasing acceptance bias), and in our exploratory dimensional finding that increasing risk of diabetes is associated with lower acceptance bias across study groups. Thus, metabolic ill-health may be associated with a blunted decision bias to expend effort for reward or, conversely, an enhanced bias to *conserve* energy.

This cognitive-computational shift appears to mimic the effects of neuropsychiatric symptoms on motivational cognition in other clinical groups. Both our previous work using the same task and modelling approach [21], as well as others’ work using different tasks but a similar modelling approach [33] find that differences in effort-based decision-making related to apathy, anhedonia, and depression, are driven by a lower acceptance bias parameter. Importantly, in our current sample, while the effect on motivation is captured by that same acceptance bias parameter, it appears *largely independent of psychiatric comorbidity*. This result requires replication, but would suggest both metabolic and mental ill-health might be associated with compromised effort-based decision-making. An exception to this was the dimensional effect of metabolic risk on effort acceptance, which remained only marginally significant when controlling for current or past psychiatric disorders, suggesting the possibility that the metabolic effect on effort-based decision-making may, in part, be driven by psychiatric comorbidity, though statistical multicollinearity in this example makes that conclusion uncertain. Overall, it is likely that metabolic and neuropsychiatric health exhibit parallel effects on effort-based decision-making, which could itself contribute to the multicollinearity we observe, that is, the known comorbidity metabolic and mental ill-health. This suggests a common neurocognitive endophenotype of metabolic and neuropsychiatric health.

The specificity of our current results, as well as previous results investigating neuropsychiatric symptoms [21, 33], to the acceptance bias parameter warrants some consideration. The effort and reward sensitivity parameters describe how strongly an individual’s decision-making adapts to *changes* in effort level or reward magnitude, respectively. The acceptance bias parameter, by contrast, functions as an intercept: a general motivational tendency towards or away from engaging in effortful behaviour, independent of the specific effort-reward tradeoff on offer. These results are somewhat at odds with previous work linking the reward sensitivity parameter specifically to dopaminergic signalling [34, 35]. However, it is worth noting that these studies employ model spaces that do not include an acceptance bias parameter, limiting comparability and posing the question whether association between dopaminergic signalling and reward sensitivity reflects a true computational specificity, or is instead a consequence of a constrained model space.

Overall, our findings align with a broader framework regarding the brain’s involvement in allostatic energy regulation [6, 8, 36]. To ensure energy balance, the brain must balance energy expenditure and conservation in coordination with internal metabolic states–dynamic regulation of motivational cognition may represent a higher-order mechanisms by which energy-expending behaviour is aligned with energy allostatic goals. A state of metabolic ill-health, as present in our samples of participants with type-2 diabetes, may be associated with apparent dysregulation of these processes. Cognitively, this may present as a bias away from effortful behaviour, potentially owing to alterations in dopamine signalling [2].

A key consideration is that the cross-sectional nature of this study means we cannot draw any conclusions about the direction of effect: are changes in motivation the consequence of metabolic ill-health, or do they precede them? Both directions of effect are conceivable and should be discussed [8]. Changes in metabolic processes may disrupt cognition, for instance through interactions between insulin and dopamine [12, 37], giving rise to the observed altered effort-based decision-making. Alternatively, biased effort-based decision-making could confer risk for poor metabolic health by altering behaviour and decisions (for example, toward conserving rather than expending energy), with downstream metabolic consequences. These different directions of effect between metabolic health and motivational decision-making are not necessarily in competition: instead, our results could be explained by a positive feedback loop wherein decreased motivational tendencies and a behavioural bias towards energy conservation inhibits behaviours such as physical exercise, leading to weight gain and the continued maintenance or progression of metabolic dysfunction, which due to biological interactions between metabolic and neurocognitive mechanisms, also alter cognition and worsen motivational tendencies.

Given semaglutide’s effects on dopaminergic signalling [38] we were surprised to find no difference in effort-based decision-making between participants on or off semaglutide. We consider two explanations for this result. First, it is possible that despite our group matching approach, sampling differences between type-2 diabetes subjects on and off semaglutide resulted in systematic differences between the groups. In support of this, exploratory analyses revealed that the semaglutide group exhibited markers of more advanced metabolic disease relative to the off-semaglutide group, including a greater number of diabetes medication classes, a higher prevalence of diabetes-relevant cardiometabolic comorbidities, and a greater cardiovascular medication burden. This pattern suggests that individuals prescribed semaglutide in our sample may represent a more severe disease phenotype, for instance, those for whom earlier lines of treatment were unsuccessful. Given the numerically lower acceptance bias parameter in the type-2 diabetes group on semaglutide, these findings align with the idea that altered effort-based decision-making tracks with metabolic ill-health in a dimensional manner, as suggested by results discussed above.

Alternatively, semaglutide treatment may genuinely have no effect on effort-based decision-making in type-2 diabetes. Studies examining the effects of GLP-1 receptor agonists on neural reward reactivity tend to report more consistent findings for acute rather than long-term administration, possibly due to neural adaptations with long-term exposure [39]. As participants in the semaglutide group in the current study had been receiving treatment for a minimum of four weeks, such adaptations may already have been established at the time of testing. Additionally, the existing literature on GLP-1 and reward processing has focused almost exclusively on food rewards. In our current study, we used points as rewards in order to tap into domain-general motivational processes, raising the possibility that effects of semaglutide on reward processing does not generalise beyond food-related stimuli. Further work, ideally a randomised controlled trial on the effects of semaglutide on effort-based decision-making, is necessary to understand its effects on motivation.

Our results should be seen in light of several (additional) limitations, the most important of which is that their cross-sectional nature precludes any assessment of a causal relationship between metabolic health and motivation. Longitudinal epidemiological studies suggest type-2 diabetes and depression have a reciprocal relationship: the presence of either condition increases the risk of subsequently developing the other [40, 41]. In future, to better understand the possible role of altered cognition and acceptance bias in this bi-directional relationship, longitudinal studies should measure changes in effort-based decision-making over time and assess how this relates to metabolic health changes.

Moreover, conducting our study online allowed us to access a much larger and more diverse population. However, a corollary of this is that we were unable to obtain physiological measures from participants, limiting us in our ability to infer underlying biological mechanisms. A laboratory experiment taking quantitative measures of metabolic health (e.g., insulin sensitivity) would be a useful complement. Based on the results presented here, we would predict participants with poorer insulin sensitivity would show a corresponding shift in acceptance bias. A further limitation concerns the characterisation of disease severity in our type-2 diabetes groups. Our study did not collect data on time since diagnosis, which would have provided a more direct index of disease duration. Instead, we relied on proxy measures of severity (medication and comorbidity burden) which, while informative, are indirect and subject to self-report bias.

In summary, we identified a putative cognitive-computational signature common to diabetes and neuropsychiatric conditions: reduced acceptance bias during motivational decision-making. The reduced motivational acceptance bias in type-2 diabetes may be the consequence of metabolic disruptions, like insulin resistance, or precede them. Importantly, we showed that while the deficit in motivational decision-making found in type-2 diabetes is parallel to that found in apathy and anhedonia, our effects were not driven by psychiatric comorbidity. Instead, altered effort-based decision-making in type-2 diabetes may represent the cognitive manifestation of a broader energy regulation phenotype. This is an important preliminary finding with relevance for metabolic-mental health comorbidity, that we hope will be explored further in studies using complementary methodologies to understand the causality, directionality, and underlying mechanisms.

## Supplementary information

Supplemental Information

## Data Availability

The analysis code and data are openly available at github.com/smehrhof/semaglutide-study. The effort-expenditure task code is available at github.com/smehrhof/effort-study. A previous version of this article has been published as a pre-print on PsyArXiv: https://doi.org/10.31234/osf.io/4bkm9. For the purpose of open access, the author has applied a Creative Commons Attribution (CC BY) licence to any Author Accepted Manuscript version arising from this submission.

## Code availability

The analysis code and data are openly available at github.com/smehrhof/semaglutide-study. The effort-expenditure task code is available at github.com/smehrhof/effort-study. A previous version of this article has been published as a pre-print on PsyArXiv: https://doi.org/10.31234/osf.io/4bkm9. For the purpose of open access, the author has applied a Creative Commons Attribution (CC BY) licence to any Author Accepted Manuscript version arising from this submission.
