# Supplementary material for: Computational phenotyping of effort-based decision-making in type-2 diabetes on and off semaglutide
Source: Neuropsychopharmacology. 2026 May 6;51(11):1924–31. doi: 10.1038/s41386-026-02433-y (PMC13597482; doi:10.1038/s41386-026-02433-y)
Supplement: Supplementary file 1 [file 41386_2026_2433_MOESM1_ESM.docx]

**Supplemental Information**

1. **Model agnostic task analyses**

To complement computational modelling analysis reported in the main text we present a series of model-agnostic analyses of participants task data. General task performance metrics per group are presented in Table S1, showing that groups did not differ significantly in the time taken to complete the task, overall clicking capacity resulting (i.e., effort calibration values), proportion of accepted but failed trials, and response times.

**Table S1.** Model agnostic task metrics between groups.

|  | **Type-2 diabetes, on semaglutide** | **Type-2 diabetes, off semaglutide** | **No diabetes, BMI matched** | **No diabetes, BMI 18.5-25** | **Significance** |
| --- | --- | --- | --- | --- | --- |
| Sample size, number (%) | 58 (25.44) | 54 (23.68) | 58 (25.44) | 58 (25.44) |  |
| Time on task in minutes, mean (SD, range) | 34.28 min. (6.84,  25.13-49.60) | 33.39 min. (11.44,  25.76-91.46) | 35.78 min. (17.41,  23.53-150.94) | 32.47 min. (5.18,  25.82-48.79) | *p =* .44 |
| Clicking capacity, mean (SD) | 60.63 (23.42) | 62.27 (13.35) | 61.91 (14.48) | 60.95 (16.85) | *p =* .95 |
| Proportion of accepted but failed trials in %,  mean (SD) | 2.19 (4.17) | 1.58 (2.59) | 1.17 (2.40) | 1.73 (4.08) | *p =* .44 |
| Reaction times,  mean (SD) | 4814.62 (3292.29) | 4126.05 (3989.50) | 5068.22 (9814.24) | 3857.54 (1709.92) | *p =* .36 |

Acceptance rates across effort and reward levels are presented in Figure S1. Note, however, a key interpretational limitation applies to the model agnostic aggregated choice data: due to the staircasing procedure used in this task, trial presentation was individualised based on each participant’s prior choices, such that not all participants received equivalent sampling of each effort-reward combination. The computational model was designed to account for this structure, and should be considered the primary basis for inference.

Group differences in acceptance rate emerge particularly in the low reward, high effort trials (Fig. 1, top left and bottom right panels), with the *Type-2 diabetes, on semaglutide* group exhibiting lowest acceptance rates and the *No diabetes, low BMI* group the highest acceptance rates.

**Figure S1. Acceptance rates across effort and reward levels, per group.** Acceptance rates across effort and reward levels, stratified by study group. The top panels show mean acceptance rates as a function of effort level (1–4, corresponding to 30%, 50%, 70%, and 90% force requirements) for low-reward (Rewards 1–2; top left) and high-reward (Rewards 3–4; top right) trials. The bottom panels show mean acceptance rates as a function of reward level across low-effort (Efforts 1–2; bottom left) and high-effort (Efforts 3–4; bottom right) trials. Error bars represent ±1 standard error of the mean.

2. **Computational modelling results**

All models considered in our model-based analysis approach converged well, as indicated by split R-hats (all < 1.001) and ESS (all > 13555) and visual inspection of trace plots. Formal model comparison by LOOIC, conducted separately for each study group, consistently identified the model implementing a parabolic cost function and including three free parameters (effort sensitivity 𝛽_𝐸_, reward sensitivity 𝛽_𝑅_, and acceptance bias 𝛼) as the best fitting model across all study groups (Fig. S2).

**Figure S2. Model comparison.** Model comparison based on leave-out-out

information criterion (LOOIC; lower is better) identified the full parabolic model as the winning model in all study groups. Error bars indicate standard errors.

Posterior predictive checks further validated the winning model in all study groups, showing a good accordance between the observed and model-predicted proportion of accepted trials (Fig. S3).

**Figure S3. Posterior predictive checks.** Posterior predictive checks for the full

parabolic model, comparing observed vs. model-predicted subject-wise acceptance

proportions across effort levels for each study group

3. **Questionnaire-based measures**

**Table S2.** Questionnaire-based measures and statistical significance of difference between type-2 diabetes study groups.

|  | **Type-2 diabetes, on semaglutide** | **Type-2 diabetes, off semaglutide** | **Significance** |
| --- | --- | --- | --- |
| **Sample size, number (%)** | 58 (51.79%) | 54 (48.21%) |  |
| **BDI, mean (SD)** | 15.9 (11.9) | 13.9 (11.2) | *p =* .359 |
| **SHAPS, mean (SD)** | 10.4 (7.71) | 8.83 (6.73) | *p =* .250 |
| **AES, mean (SD)** | 54.4 (10.7) | 55.1 (11.0) | *p =* .735 |
| **OCI-R, mean (SD)** | 15.5 (11.4) | 15.8 (12.8) | *p =* .891 |
| **MCQ, mean (SD)** | 0.029 (0.030) | 0.027 (0.030) | *p =* .703 |
| **DAQ, mean (SD)** | 9.97 (5.95) | 10.9 (7.37) | *p =* .443 |
| **TFEQ, mean (SD)** |  |  |  |
| **Cognitive restrained** | 17.3 (2.48) | 17.8 (2.21) | *p =* .333 |
| **Uncontrolled eating** | 22.6 (4.09) | 23.9 (4.00) | *p =* .086 |
| **Emotional eating** | 6.83 (2.42) | 7.42 (2.37) | *p =* .189 |
| **MCTQ, mean (SD)** | 280 (131) | 312 (166) | *p =* .269 |
| **MEQ, mean (SD)** | 48.2 (12.5) | 46.7 (12.6) | *p =* .505 |

*Note.* SD, Standard deviation; BDI, Beck Depression Inventory; SHAPS, Snaith-Hamilton Pleasure Scale; AES, Apathy Evaluation Scale; OCI-R, Obsessive-Compulsive Inventory – Revised; MCQ, Monetary Choice Questionnaire; DAQ, Desire for Alcohol Questionnaire; TFEQ, Three-factor Eating Questionnaire; MCTQ, Munich Chronotype Questionnaire; MEQ, Morningness-Eveningness Questionnaire.

4. **Disease severity proxies in type-2 diabetes groups**

To explore whether the two type-2 diabetes groups differed in disease severity, we conducted exploratory descriptive comparisons on three proxies: number of diabetes medication classes, number of diabetes-relevant physical comorbidities, and cardiovascular medication burden.

The type-2 diabetes group on semaglutide were also taking a greater number of diabetes medication classes (On semaglutide: 51 participants (87.931%) on >1 diabetes medication classes; Off semaglutide: 10 participants (18.519%) on >1 diabetes medication classes; Figure S4 left), had a higher number of diabetes-relevant physical comorbidities (On semaglutide: 9 participants (15.517%) with >0 comorbidity; Off semaglutide: 6 participants (11.111%) with >0 comorbidity; Figure S4 middle), and carried a greater cardiovascular medication burden (On semaglutide: 34 participants (58.621%) on >0 cardiovascular medication; Off semaglutide: 23 participants (42.593%) on >0 cardiovascular medication; Figure S4 right) compared to the *type-2 diabetes, off semaglutide* group.
Tables S3-S5 provide a full breakdown of the specific medication classes and comorbidities recorded across both groups.

**Figure S4. Posterior predictive checks.** Posterior predictive checks for the full

parabolic model, comparing observed vs. model-predicted subject-wise acceptance

proportions across effort levels for each study group

**Table S3.** Number of diabetes medication classes between type-2 diabetes study groups.

|  | **Type-2 diabetes, on semaglutide (n=58)** | **Type-2 diabetes, off semaglutide (n=54)** |
| --- | --- | --- |
| **GLP-1 agonists** | 58 (100%) | 0 (0%) |
| **Biguanides** | 39 (67.241%) | 36 (66.667%) |
| **Insulin** | 17 (29.310%) | 4 (7.407%) |
| **SGLT2 inhibitors** | 10 (17.241%) | 3 (5.556%) |
| **Sulfonylureas** | 8 (13.793%) | 7 (12.963%) |
| **Thiazolidinediones** | 4 (6.897%) | 1 (1.852%) |
| **DPP-4 inhibitors** | 0 (0%) | 2 (3.704%) |
| **Meglitinides** | 0 (0%) | 1 (1.852%) |
| **Alpha-glucosidase inhibitors** | 0 (0%) | 1 (1.852%) |

*Note.* SGLT2, Sodium-glucose transporter 2; DPP-4, Dipeptidyl peptidase-4.

**Table S4.** Number of diabetes-relevant physical comorbidities between type-2 diabetes study groups.

|  | **Type-2 diabetes, on semaglutide (n=58)** | **Type-2 diabetes, off semaglutide (n=54)** |
| --- | --- | --- |
| **Heart disease** | 6 (10.345%) | 2 (3.704%) |
| **Kidney disease** | 5 (8.621%) | 2 (3.704%) |
| **Hypertension** | 1 (1.724%) | 1 (1.852%) |
| **Stroke** | 1 (1.724%) | 1 (1.852%) |
| **Liver disease** | 1 (1.724%) | 1 (1.852%) |

**Table S5.** Cardiovascular medication between type-2 diabetes study groups.

|  | **Type-2 diabetes, on semaglutide (n=58)** | **Type-2 diabetes, off semaglutide (n=54)** |
| --- | --- | --- |
| **Antihypertensives** | 27 (46.552%) | 18 (33,333%) |
| **Statins/lipid-lowering** | 26 (44.828%) | 18 (33,333%) |
| **Anticoagulants/antiplatelets** | 3 (5.172%) | 4 (7.407%) |
